# STAT3 enhances the constitutive activity of AGC kinases in melanoma by transactivating PDK1

**DOI:** 10.1186/s13578-018-0265-8

**Published:** 2019-01-03

**Authors:** María Elisa Picco, María Victoria Castro, María Josefina Quezada, Gastón Barbero, María Belén Villanueva, Natalia Brenda Fernández, Hyungsoo Kim, Pablo Lopez-Bergami

**Affiliations:** 1 Instituto de Medicina y Biología Experimental (IBYME), Consejo Nacional de Investigaciones Científicas y Técnicas (CONICET), Buenos Aires, Argentina; 2 Centro de Estudios Biomédicos, Biotecnológicos, Ambientales y Diagnóstico (CEBBAD), Universidad Maimónides, CONICET, Hidalgo 775, 6th Floor, Lab 602, Buenos Aires, Argentina; 3 Sanford Burnham Prebys Medical Discovery Institute, La Jolla, CA USA

**Keywords:** STAT3, PDK1, Akt, PKC, SGK, Melanoma

## Abstract

**Background:**

The PI3K/Akt and the STAT3 pathways are functionally associated in many tumor types. Both in vitro and in vivo studies have revealed that either biochemical or genetic manipulation of the STAT3 pathway activity induce changes in the same direction in Akt activity. However, the implicated mechanism has been poorly characterized. Our goal was to characterize the precise mechanism linking STAT3 with the activity of Akt and other AGC kinases in cancer using melanoma cells as a model.

**Results:**

We show that active STAT3 is constitutively bound to the PDK1 promoter and positively regulate PDK1 transcription through two STAT3 responsive elements. Transduction of WM9 and UACC903 melanoma cells with STAT3-small hairpin RNA decreased both PDK1 mRNA and protein levels. STAT3 knockdown also induced a decrease of the phosphorylation of AGC kinases Akt, PKC, and SGK. The inhibitory effect of STAT3 silencing on Akt phosphorylation was restored by HA-PDK1. Along this line, HA-PDK1 expression significantly blocked the cell death induced by dacarbazine plus STAT3 knockdown. This effect might be mediated by Bcl2 proteins since HA-PDK1 rescued Bcl2, Bcl-XL, and Mcl1 levels that were down-regulated upon STAT3 silencing.

**Conclusions:**

We show that PDK1 is a transcriptional target of STAT3, linking STAT3 pathway with AGC kinases activity in melanoma. These data provide further rationale for the ongoing effort to therapeutically target STAT3 and PDK1 in melanoma and, possibly, other malignancies.

**Electronic supplementary material:**

The online version of this article (10.1186/s13578-018-0265-8) contains supplementary material, which is available to authorized users.

## Background

The transcription factor Signal Transducer and Activator of Transcription 3 (STAT3) shows low or null activity in normal unstimulated cells but an enhanced activity in various types of human cancer cells. Compelling evidence has established that aberrant STAT3 activity has a critical role in the development and progression of human tumors by promoting uncontrolled cell proliferation and growth, cell survival, induction of angiogenesis, and the suppression of host immune surveillance [1, 2].

Melanoma is a highly aggressive skin cancer whose incidence has been rising substantially over the last few decades worldwide [3]. If diagnosed early, melanoma is curable by surgical resection. However, the prognosis of metastatic melanoma is poor with a 5-year survival rate lower than 20%. Malignant melanoma is a difficult cancer to treat given its resistance to chemotherapy and radiotherapy [4]. Targeting of the prevalent BRAF V600E mutation (present in around 50% of patients) with vemurafenib or similar compounds produce clinical responses in most melanoma patients but all patients develop resistance and relapse, highlighting the need of new therapeutic targets [5].

A large body of evidence has implicated hyperactive receptor tyrosine signaling in the development and progression of melanoma. These include mutations on KIT, ERBB4, the EPH and FGFR families, genomic amplification of EGFR and PDGRFA among others [6]. Therefore, it is not surprising that STAT3, being a point of convergence of many of these signaling pathways, has been found to be activated at high frequency and has been implicated in melanoma progression [7–9]. The levels of p-STAT3 is higher in metastasis (particularly brain and lung) than in cutaneous primary melanomas [9, 10]. Also, p-STAT3 expression is a negative prognostic factor for overall survival in patients that did not develop central nervous system metastasis [9, 10]. Recent pieces of evidence have shown that STAT3 activation is an important mechanism of resistance to targeted therapies against mutant BRAF, a critical oncogene in melanoma [11–14]. Many of the above-mentioned alterations result in a persistent phosphorylation of STAT3 at Tyrosine 705 (Y^705^) and STAT3-dependent transactivation of target genes through binding of STAT3 dimers to consensus STAT3 binding sequences on their promoters [15]. A large number of genes whose transcription is regulated by STAT3 have been identified (i.e. Cyclin D1/D2, c-Myc, p21WAF, VEGF, Mcl-1 and Bcl-xL) [16]. However, evidence from microarray and ChIP-seq studies have revealed that a large number of potential STAT3 target genes remains to be characterized [17–20]. Since STAT3 is emerging as a target of interest for many cancers it is essential to identify novel STAT3 target genes that will help understand the pleiotropic functions of STAT3 in tumorigenesis.

In the present work, we studied a new transcriptional target of STAT3, phosphoinositide-dependent kinase 1 (PDK1). PDK1 is the master regulator of at least 23 other AGC kinases whose downstream signaling has often been implicated in various diseases and particularly in cancer [21]. The AGC is a large group of protein kinases (more than 60) named after the protein kinase A, G, and C families (PKA, PKG, PKC). To be active, these kinases require phosphorylation at both the activation loop and the hydrophobic domain (e.g. Thr^308^ and Ser^473^ for Akt1, by PDK1 and mTORC2, respectively) [22]. Several mechanisms contribute to the constitutive activity of many AGC kinases in melanoma. For example Akt, one of the prominent members of this family, is constitutively activated by the concurrent effect of Ras mutations or PTEN loss, Akt amplification and active-functioning autocrine loops [8]. PDK1 itself has been recognized to have an important role in cancer [22]. In melanoma, PDK1 has been implicated in disease development and progression through regulation of Akt, SGK3 and FOXO3a [23, 24]. For this reason, great efforts have been dedicated to understanding how PDK1 itself is regulated and how, in turn, regulates its different substrates spatially and temporally. Unlike the other kinases in the AGC family, PDK1 is autophosphorylated [25] meaning that, for the most part, its function is not regulated by an upstream kinase but by alternative mechanisms like protein–protein interaction, conformational rearrangements, changes in subcellular localization or at the transcriptional level [26, 27]. The present work describing the STAT3-dependent regulation of PDK1 offers important new insights into the mechanism underlying STAT3 oncogenic activity through feeding into AGC kinase activity.

## Methods

### Cell culture

Melanoma lines were kindly provided by Dr. M. Herlyn and Dr. Z. Ronai. All cell lines were maintained in DMEM supplemented with 10% fetal bovine serum (FBS, Gibco) 100 U/ml penicillin and 100 mg/ml streptomycin (Invitrogen), at 37 °C and 5% CO_2_. Cell lines were validated in 2016 by short tandem repeat analysis (STR) of extracted genomic DNA using GenePrint 10 System, Promega, according to manufacturer instructions. Cells were transfected with calcium phosphate or by Lipofectamine PLUS reagent (Invitrogen) following the manufacturer’s protocol.

### Viral constructs

The oligonucleotides targeting STAT3 (5′-GCAGCAGCTGAACAACATG-3′ and 5′-GATTGACCTAGAGACCCAC-3′) [28] and scramble (5′-GAAACTGCTGACCGTTAAT-3′) were cloned into pRetroSuper vector. To generate the viral particles, HEK-293T producer cells were cotransfected with the retro vectors and the packaging plasmids. Viral supernatants were harvested, filtered and used to transduce WM9 and UACC903 cells. Cells were selected in 3 µg/ml puromycin for 1 week and then maintained with 1 µg/ml puromycin. WT PDK1 cDNA together with a HA tag was cloned into pBABE-Hygro as described [26]. This vector was introduced into WM9 cells in the same manner and transduced cells were selected with 100 μg/ml Hygromycin B (Gibco).

### Real time PCR

Real time PCR was performed as described [27]. Specific primers used for PCR were as follows: PDK1 forward 5′GTCTTATCCCCAGAGAGCAAAC3′; PDK1 reverse, 5′AGCAGCTCTGGAGAAACGTACT3′, RNPII forward 5′GCTGTGTCTGCTTCTTCTG3′, RNPII reverse 5′CGAACTTGTTGTCCATCTCC3′ RNA polymerase II (RNPII) served as an endogenous control. Reactions were run in triplicate. The target mRNA concentration of control cells, normalized to the level of RNPII mRNA, was set to 1.

### Chromatin immunoprecipitation (ChIP)

For ChIP analysis, WM9 cells were fixed with 11% formaldehyde and sheared chromatin was immunoprecipitated with a STAT3 antibody (sc-482, Santa Cruz Biotechnology) or control IgG and subjected to real-time PCR. The following primers corresponding to the proximal region of the PDK1 promoter were used: PDK1 forward 5′GAGCCTGGTCCCCTCTGA3′ and PDK1 reverse 5′GATTGGTTCGCGCGAGGT3′.

### Trypan blue exclusion assay

The WM9 cells were seeded on 24-well plates at a density of 4 × 10^4^ cells per well and incubated for 24 h to adhere. Cells were exposed to 100 μg/ml dacarbazine (dissolved in PBS) or PBS as a control for 72 h. After that time point, both the adherent and floating cells were collected, mixed with trypan blue and the number of viable cells was counted using a haemocytometer. The percentage of non-viable cells regarding the total number of cells was calculated. Each treatment was performed in duplicate and three independent experiments were performed. Error bars represent the standard errors.

### Resazurin assay

To study relative cell viability, 3000 cells per well were seeded (in 100 μl volume of DMEM minus phenol red) in 96-well microplates in eight-duplicates. After 24 h, cells were treated with 100 μg/ml dacarbazine for 72 h. Then, Rezasurin (Cayman Chemical), the active compound of the commercially available dye Alamar Blue (Bio-Rad), was added at a final concentration of 0.4 mM and the plates were incubated for 4 h at 37C. The absorbance at 570 nm and 600 nm was determined in a Biotek plate reader. A standard curve with different cell densities was prepared in a parallel plate. To determine the differences in cell viability between treated and control cells we followed the calculations described by the Alamarblue cytotoxicity assay kit (Bio-Rad).

### Quantification of apoptotic cell death

The WM9 and UACC903 cells were seeded on 6-well plates at a density of 1.25 × 10^5^ cells per well. The following day they were exposed to 100 μg/ml Dacarbazine (or PBS as a control) for 72 h. Cells were washed twice with PBS and resuspended in 100 µl of Annexin V binding buffer (pH 7.4) (BD Biosciences, Franklin Lakes, NJ, USA). Then, Annexin V-Alexa Fluor 488 (BD Biosciences) was added and incubated for 15 min under dark conditions. Propidium iodide (0.1 µg/ml; Sigma-Aldrich; Merck KGaA, Darmstadt, Germany) was added just prior to signal acquisition. Cells were analyzed using a FACSAria flow cytometer (BD Biosciences) and analyzed with FACSDiva 7.6.1 software (BD Biosciences).

### Flow cytometry

Single-cell suspensions were fixed (4% PFA) and permeabilized (PBS, 0.5% saponin, 10% FBS). Cells were incubated with primary antibodies or respective isotype controls for 30 min at 4 °C. Unconjugated Antibodies to PDK1 (3062), P-Akt (Thr308, 9275 and 2965), Akt (2967 and 9272), pan-P-PKC (9371) from Cell Signaling and Akt (sc-5298) and PKCβ (sc-210), from Santa Cruz Biotechnologies were used. Preimmune rabbit sera or isotype matched mouse Ig were used as a control. FITC-conjugated goat anti-mouse or anti-rabbit Ig was added for 30 min at RT in the dark. Cells were acquired on a FACSAria flow cytometer (BD Biosciences) and analyzed using Cyflogic software. The protein level is quantified as the mean fluorescence intensity (MFI) after subtraction of control Ab MFI.

### Luciferase assays

The PDK1 promoter reporter plasmids were generated as described [26]. Site-directed mutagenesis of STAT3 sites were performed using the QuikChange II kit (Stratagene) following the manufacturer’s protocol. Cell lysates were prepared from lipofectamine-transfected cells after 24 or 48 h. Luciferase activity was measured with the luciferase assay system (Promega) in a luminometer and was normalized with β-galactosidase activity. Results are shown as the mean (bar) ± S.D.

### Western blotting

For the Western blotting analysis, cell lysates were collected by addition of lysis buffer supplemented with protease and phosphatase inhibitors for 10 min on ice [29]. The cell lysates were centrifuged at 13,000 rpm for 15 min at 4 °C, and the supernatants were collected and quantified using the Bradford method. Between 20 and 50 μg of proteins were diluted in 6× Laemmli buffer, boiled at 95 °C for 5 min, separated on 10–12% SDS-PAGE gels and then transferred to nitrocellulose membrane. The membranes were blocked with 5% milk in 0.05% Tween-PBS at room temperature for 1 h and then incubated with the primary antibodies at 4 °C overnight. The following antibodies were used: Akt1 (sc-5298), p-Akt (sc-7985), GAPDH (sc-25778), STAT3 (sc-482), pSTAT3 (sc-8059), PKCb (sc-210), Vimentin (sc-73614), Mcl-1 (sc-819) and Bcl-XL (sc-634) from Santa Cruz Biotechnologies. Antibodies to PDK1 (3062), P-PDK1 (3061), P-Akt (Thr308, 9275 and 2965), Akt (2967 and 9272), pan-P-PKC (9371), pSGK (T256, 2939), SGK (12103) and Bcl-2 (2876) were from Cell Signaling. Antibodies to Actin (A5441) and Tubulin (T9026) were from Sigma. Because PKC autophosphorylation at the hydrophobic site depends on the efficiency of activation loop phosphorylation by PDK1, we used the P-PKC (Ser660) antibody to monitor activation loop phosphorylation. The corresponding HRP-conjugated secondary antibodies: anti-mouse (GE NA931V), anti-rabbit (GE NA934) or anti-goat (sc-2020) were incubated for 1 h at room temperature. Immunoreactive bands were detected by an ECL system (Amersham Biosciences) using an image reader (ImageQuant 350, GE Healthcare). Quantification of band intensities was performed using ImageJ (NIH). The intensity of each band was normalized to GAPDH or other housekeeping gene (i.e. Tubulin or Actin) and the Fold Change (FC) relative to control cells was calculated. The band intensities in the phosphoprotein blots were normalized with those of the total proteins obtained from the same blots after stripping and reprobing. To draw conclusion on a particular experiment at least three biological (independent) replicates of paired samples were examined to calculate the mean and standard deviation. The log transformation of FC values were calculated to obtain a more symmetric distribution that better suits the normality assumptions of the subsequent t-test.

### Statistics

Except when indicated, experiments were performed at least three times. All data are expressed as the mean ± SD. Mean differences between groups were determined using Student’s T tests or one-way ANOVAs followed by post hoc tests. Values of p < 0.05 were considered statistically significant. Statistical analyses were conducted using software from Graph-Pad Prism. The number of independent experiments and specific statistical analyses used in each experiment are indicated in the figure legends.

## Results

### STAT3β negatively regulates PDK1 expression

**Fig. 1:**
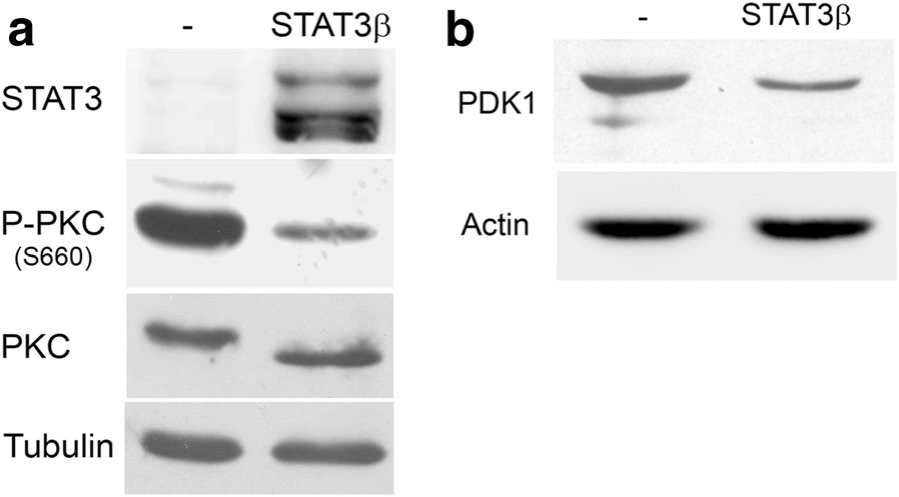
STAT3β negatively regulates PKC phosphorylation and PDK1 expression. Protein extracts from SW1 cells stably transfected with STAT3β or empty vector (−) were blotted with P-PKC and PKCβII antibodies (**a**) and PDK1 antibodies (**b**). Tubulin and actin were used as loading control. The blots displayed are representative of three independent experiments

During experiments addressing STAT3’s role in tumorigenesis, we observed that SW1 mouse melanoma cells transduced with STAT3β presented a marked reduction in the levels of PKC phosphorylated at the hydrophobic motif (Ser^660^ in PKCβII) (Fig. 1a). STAT3β is a STAT3 splice variant usually seen as a natural dominant negative form [30]. Since the hydrophobic motif of PKC is autophosphorylated subsequently to phosphorylation on the activation loop (Thr^500^ in PKCβII) by PDK1 [31], we analyzed changes in PDK1 levels in these cells. As shown in Fig. 1b, expression of STAT3β markedly decreased the expression of PDK1 (Fig. 1b). These data suggest that STAT3β might interfere with a positive regulation of PDK1 expression by STAT3.

### STAT3 regulation of PDK1 modulates phosphorylation of AGC kinases

**Fig. 2:**
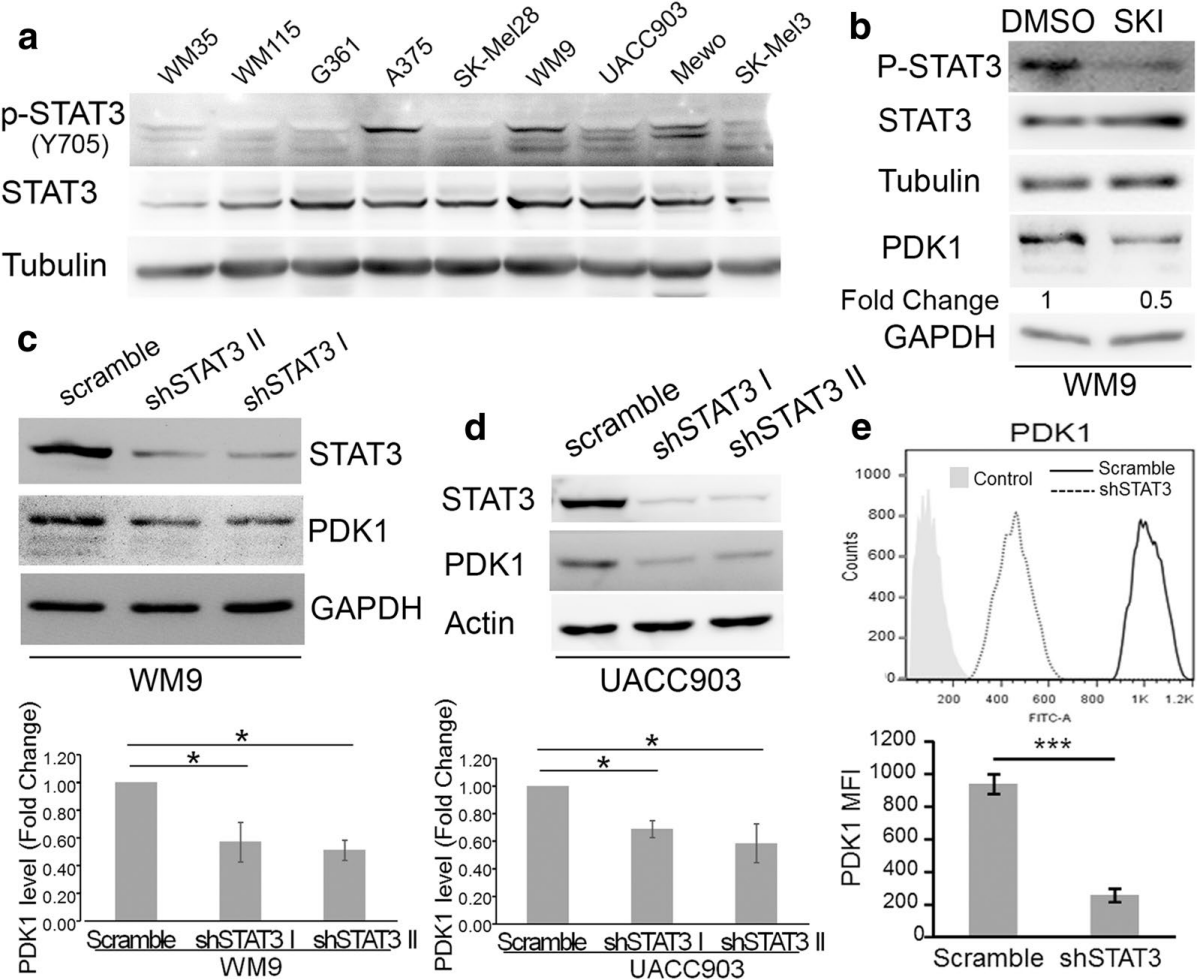
Silencing of STAT3 reduced PDK1 expression. **a** Activation of STAT3 in melanoma cell lines. Western blot analysis of STAT3 and P-STAT3 levels in human melanoma cell lines. **b** STAT3 inhibition by SKI-606 reduced PDK1 expression. WM9 cells were treated with 1 uM SKI-606 (SKI) for 8 h. Protein extracts were blotted with the indicated antibodies. PDK1 levels were normalized to the loading control and expressed as the fold change (FC, numbers below the blots) relative to DMSO-treated cells. **c**, **d** Silencing of STAT3 in WM9 (**c**) and UACC903 (**d**) cells reduced PDK1 expression. Cells were transduced with retrovirus encoding two STAT3 shRNA (I and II) and a scramble sequence as a control. Protein extracts were probed with STAT3, PDK1 and GAPDH or Actin as a loading control. The blots displayed are representative of three independent experiments. Bar graphs show the mean ± S.D. (from three independent experiments) of PDK1 levels normalized to the loading control and expressed as the fold change (FC) relative to scramble cells. Statistical significance was tested by one-tailed Student’s T-Test using log transformed FC values. *p < 0.01, n = 3. **e** Flow cytometry analysis of PDK1 expression. Cells were collected and stained as described in “Methods”. Light-grey filled histogram correspond to control (preimmune) Ab and open histograms to PDK1 ab. The MFI (Mean Fluorescence Intensity) was detected by flow cytometry. Bar graph shows the mean PDK1 MFI ± S.D. (from three independent experiments). Statistical significance was tested by a one-tailed Student’s T-Test. ***p < 0.0001, n = 3. The blots and histogram displayed are representative of three independent experiments

To study this mechanism in human melanoma cells using a suitable model we screened several melanoma cell lines to identify those displaying constitutive STAT3 activation. Phosphorylation of Tyr^705^, a marker of STAT3 activation, was detected in several cell lines (Fig. 2a), regardless of BRAF status, since all the cell lines except for Mewo are mutant for BRAF. We then used the tyrosine kinase inhibitor SKI-606 to inhibit STAT3 phosphorylation in WM9, one of the cell lines that showed constitutive STAT3 activity (Fig. 2b). Similar to what we observed in mouse cells, inhibition of STAT3 activity using SKI-606 resulted in a decrease on PDK1 levels (Fig. 2b). To further explore the regulation of PDK1 by STAT3 we employed RNA interference (RNAi) to knockdown STAT3 expression in cultured cell lines. To rule out nonspecific effects of the technique, two STAT3 specific pRetroSuper-based shRNAs (short-hairpin RNA) labeled shSTAT3-I and -II, were transduced into both WM9 and UACC903 cells. As a control, cells were transduced with a shRNA containing a scramble sequence. Western blots showed that the two shRNA very efficiently knockdown STAT3 expression (Fig. 2c, d). In agreement with our previous results, STAT3 silencing in WM9 and UACC903 cells induced a decrease in PDK1 protein levels (Fig. 2c, d). The decrease in PDK1 was statistically significant as demonstrated by quantification of Western blots from three independent biological replicates (Fig. 2c, d). Flow cytometry analysis confirmed a significantly lower expression of PDK1 in UACC903 cells upon STAT3 silencing relative to control cells (Fig. 2e).

**Fig. 3:**
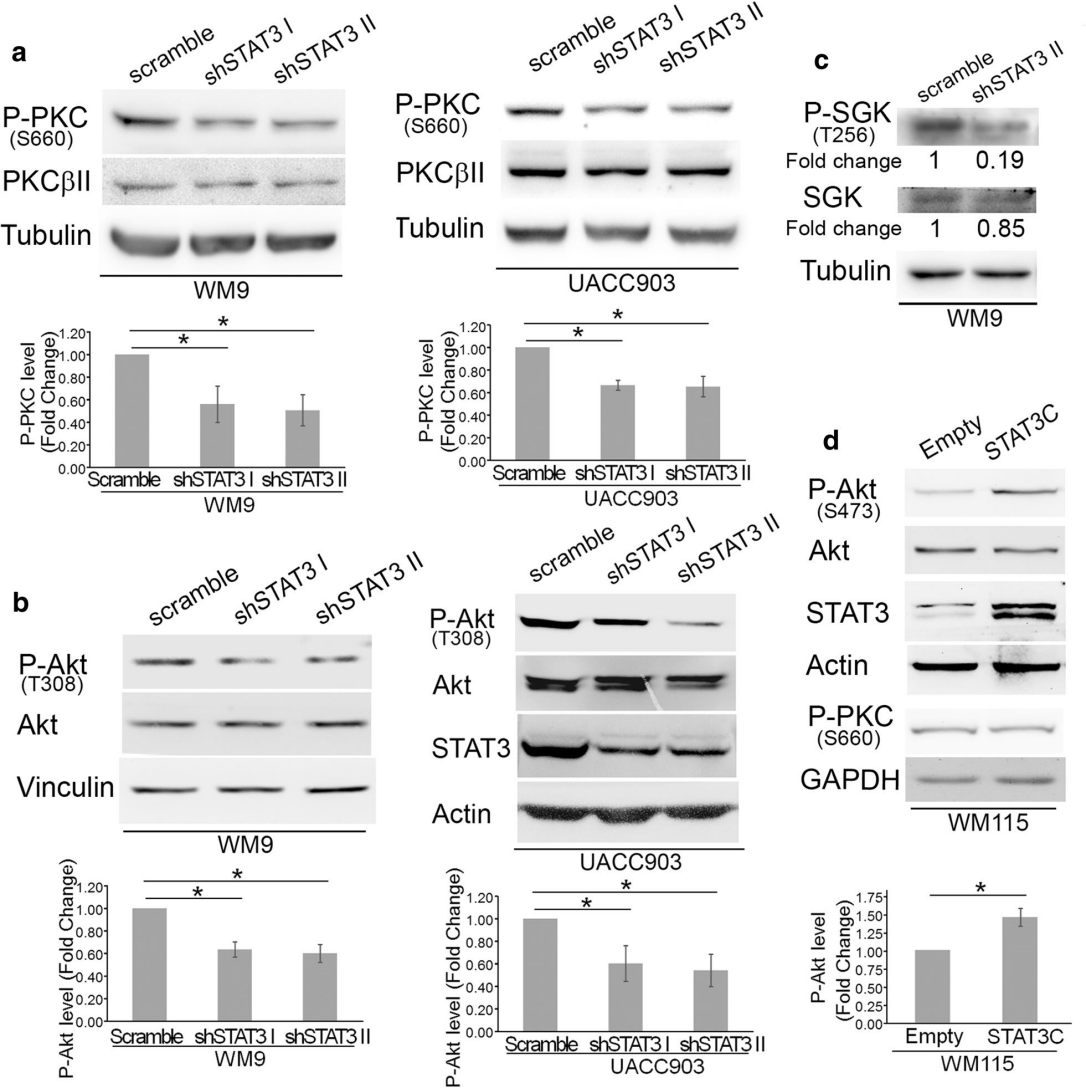
STAT3 silencing reduced PKC, Akt, and SGK phosphorylation. Protein extracts from WM9 and UACC903 cells described in Fig. 2. were assayed in Western blot to determine PKC **a**, Akt **b** and SGK **c** phosphorylation. **a**, **b** A P-Pan-PKC antibody against Ser660 was used. Total PKC was estimated by using a PKCβII antibody. Tubulin, Vinculin and Actin were used as loading controls. The blots displayed are representative of three independent experiments. Bar graphs show the mean ± S.D. (from three independent experiments) of P-PKC **a** and P-Akt **b** levels normalized to the corresponding total protein level and expressed as the fold change relative to scramble cells. Statistical significance was tested by one-tailed Student’s T-Test using log transformed FC values. *p < 0.01, n = 5. **c** WM9 extracts described above were probed with the indicated antibodies. Protein levels after STAT3 silencing were normalized to the corresponding total protein level and expressed as the fold change (numbers below the blots) relative to scramble cells. The blots displayed are representative of two independent experiments. **d** STAT3C increased P-Akt levels. Protein extracts from WM115 cells stably transfected with STAT3C were probed with the indicated antibodies. GAPDH was used as a loading control. Bar graph shows the mean ± S.D. (from three independent experiments) of P-Akt levels normalized to the corresponding total protein level and expressed as the fold change relative to Empty cells. Statistical significance was tested by one-tailed Student’s T-Test using log transformed FC values. *p < 0.01, n = 3

**Fig. 4:**
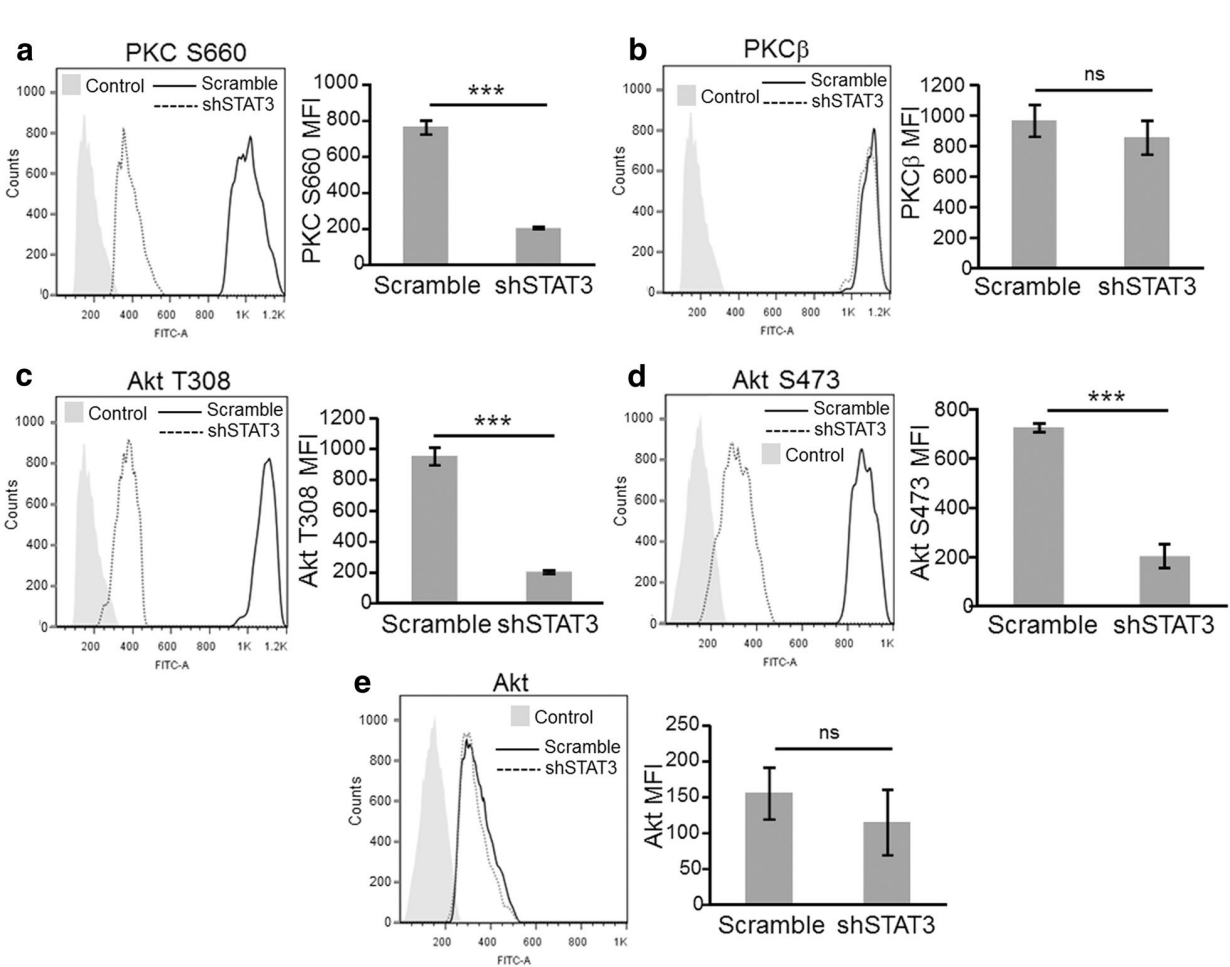
Flow Cytometry analysis of total and activated PKC and Akt upon STAT3 silencing. **a** Flow cytometry analysis of P-PKC **a**, PKCβ **b**, P-Akt (T308) **c**, P-Akt (S473) **d** and Akt **e** expression. Cells were collected and stained as described in “Methods”. Light-grey filled histogram correspond to control Ab and open histograms to the corresponding specific abs. Solid and broken lines correspond to scramble and shSTAT3 cells, respectively. Bar graphs show the mean mean fluorescence intensity (MFI) ± S.D. (from three independent experiments) for the indicated antibody. Statistical significance was tested by a one-tailed Student’s T-Test, ***p < 0.0001, ns: not significant, n = 3

As seen in mouse cells, inhibition of STAT3 in both WM9 and UACC903 cell lines significantly reduced P-PKC levels, as established by quantification of Western blot data (Fig. 3a). Since PDK1 is a master kinase that regulates the activity of several protein kinases we evaluated whether STAT3-dependent regulation of PDK1 affected phosphorylation of other AGC family kinases such as Akt and SGK. STAT3 knockdown significantly decreased phosphorylation of Thr^308^ of Akt, an indispensable event in Akt activation [31] in both UACC903 and WM9 cell lines as determined by quantitative analysis of five biological replicas by Western blot (Fig. 3b). Total Akt levels were not affected. Likewise, STAT3 silencing prevented SGK activation by inhibiting phosphorylation of Thr^256^ at its activation loop (Fig. 3c). Although the decrease in P-PKC and P-Akt levels was significant and consistently observed, we wanted to further document these changes using a fully quantitative and more sensitive technique such as Flow cytometry. The analysis performed confirmed that STAT3 silencing significantly reduced the level of P-PKC (Ser660) and P-Akt (Thr308) in UACC903 cells (Fig. 4a, c). In these experiments we also determined that phosphorylation of Akt at Ser473 was also significantly inhibited by STAT3 knockdown (Fig. 4d), confirming the impairment of the Akt activity. This technique also confirmed that total Akt and PKCβ levels were not affected by STAT3 silencing (Fig. 4b, e). To confirm these findings using an alternative approach we stably transfected WM115 cells (that do not present STAT3 phosphorylation, Fig. 2a) with STAT3C, a constitutively active STAT3 mutant with two Cys substitutions that enable STAT3 molecules to dimerize spontaneously without phosphorylation at Y705 [1]. In agreement with previous data, expression of STAT3 induced an increase in P-Akt (Ser473) levels (Fig. 3d). We did not observed changes in P-PKC (Pan) in these cells. Altogether, these results indicate that STAT3 regulates PDK1 expression and modulates Akt, PKC and SGK activity.

### STAT3 binds human PDK1 promoter and activates PDK1 transcription

**Fig. 5:**
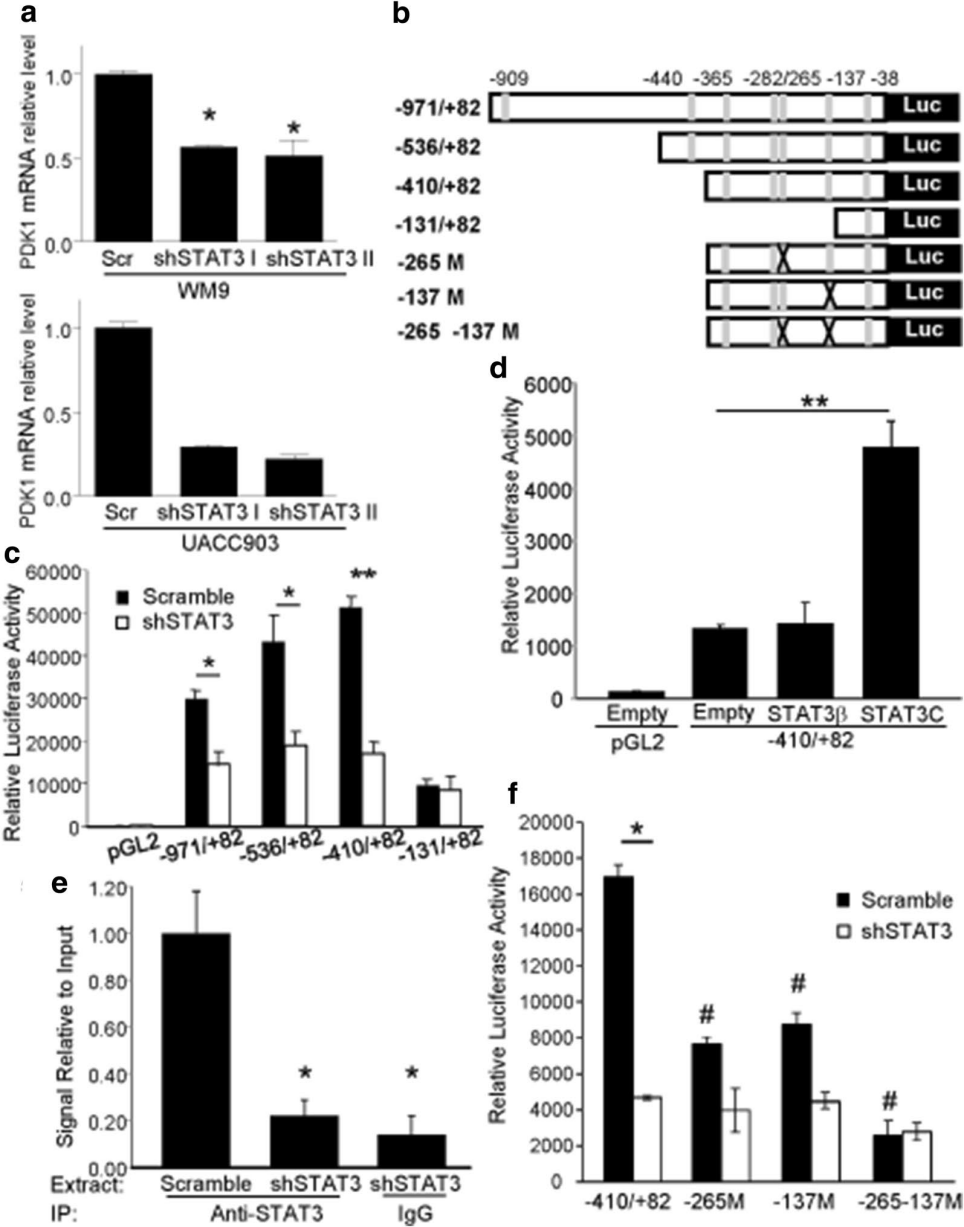
STAT3 binds to the PDK1 promoter and regulates its transcription. **a** STAT3 silencing decreased PDK1 mRNA levels. Relative levels of PDK1 mRNA were determined by Real-Time PCR in both UACC903 and WM9 cells stably transduced with two STAT3 shRNA targeting sequences. mRNA levels were normalized to internal RNPII levels and expressed as relative to control cells. For WM9 cells, the mean ± S.D. from three independent experiments is shown. *p < 0.05. For UACC903 cells, the mean ± S.D. from one representative experiment out of two is shown. **b** Structure of the proximal region of the human PDK1 promoter. Putative STAT3 responsive elements (in grey) and fragments of the promoter that were cloned into pGL2 are depicted. The sites at − 265 and − 137 were removed by mutagenesis. **c** Silencing of STAT3 diminishes the reporter activity of the PDK1 promoter. The indicated reporter plasmids were transfected into UACC903-shSTAT3 and UACC903-scramble cells. Results are shown as the mean ± S.D. *p < 0.05. **p< 0.01. **d** Active STAT3 induced luciferase activity driven by the PDK1 promoter. The plasmid − 410/+ 82 was co-transfected into WM115 cells together with STAT3β and STAT3C plasmids. Results are shown as the mean ± SD. **p < 0.01. **e** STAT3 silencing decrease binding of STAT3 to the PDK1 promoter. The plot shows the relative level of PDK1 amplification (normalized to GAPDH levels) following a Chromatin immunoprecipitation assay on UACC903-shSTAT3 and UACC903-scramble cells. The mean ± S.D. from three independent experiments is shown. *p < 0.05. **f** STAT3 enhances PDK1 transactivation through the SREs at − 265 and − 137. Reporter plasmids described in **b** were transfected into UACC903-shSTAT3 and UACC903-scramble cells. Results are shown as the mean ± S.D. *p < 0.001, Student’s t-Test; the activity of the mutant promoters was compared to that of − 410/+ 82 fragment. #p < 0.001, ANOVA followed by Dunnett’s Multiple Comparison Test

The data above showed a reduction of PDK1 protein levels in STAT3 deficient cells. Real-Time PCR experiments revealed that STAT3 silencing in both WM9 and UACC903 cells also led to a significant decrease in PDK1 mRNA levels (Fig. 5a) suggesting that STAT3 could regulate PDK1 levels through direct transactivation of its promoter. To study this possibility we performed in silico analysis of the DNA sequence 1-kb upstream of the human PDK1 transcription start site, a region that present Histone 3 modification patterns typical of enhancer elements (UCSC genome browser, data not shown). In this region, we looked for putative STAT3 responsive elements (SRE) using ECR Browser and rVista 2.0, loaded with the Transfac 10.2 matrix library [32]. Using a matrix similarity cut-off of 0.9, the analysis found 7 potential SRE starting at positions − 909, − 440, − 365, − 282, − 265, − 137 and − 38 of the human PDK1 promoter. Thus, we cloned this region of the human PDK1 promoter (1053 bp) into a luciferase reporter plasmid and generated a series of truncated constructs that are represented in Fig. 5b. After transfection into UACC903 control cells (UACC903-scramble), the three larger promoter fragments (− 971/+ 82, 536/+ 82 and − 410/+ 82) displayed a high degree of activity (more than 300 times) over the baseline control construct containing only the luciferase gene (Fig. 5c). Among the three larger promoter fragments, 971/+ 82 and − 410/+ 82 showed the lowest and higher promoter activity, respectively. Importantly, the three promoter fragments elicited a reduced luciferase activity when transfected into UACC903 stably expressing STAT3 shRNA (UACC903-shSTAT3) compared to the scramble control (UACC903-scramble) (Fig. 5c). On the contrary, the shortest promoter fragment (− 131/+ 82) showed similar luciferase activity both in UACC903-shSTAT3 and UACC903-scramble cells. Similar results were obtained when the reporter plasmids were cotransfected with STAT3β (Additional file 1: Figure S1). The similar response elicited by the three larger promoter fragments suggest the functional SRE is situated in the region between nucleotides − 410 and − 131. These experiments could not be performed in WM9 cells since these cells are poorly transfectable. We next examined whether increased levels of activated STAT3 induce luciferase activity driven by the PDK1 promoter. To investigate this possibility, we cotransfected WM115 cells with the − 410/+ 82 fragment together with STAT3C. Figure 5d shows that transfection with STAT3C but not STAT3β, increased reporter activity driven by the − 410/+ 82 fragment (Fig. 5d). These results further point to the presence of one or more SRE in the PDK1 promoter region from − 410 to − 131.

Further support for the role of STAT3 in the regulation of PDK1 transcription comes from ChIP analysis. To this end, sheared chromatin was immunoprecipitated with antibodies to STAT3 (or control IgG) followed by Real-Time PCR amplification of PDK1 promoter sequences bearing putative SRE. Immunoprecipitation of STAT3 enabled amplification of a DNA fragment corresponding to a region from − 300 to − 177 of the PDK1 promoter, demonstrating in vivo binding of STAT3 to the PDK1 promoter. Silencing of STAT3 reduced the amount of DNA amplified from the chromatin after STAT3 immunoprecipitation to values similar to those observed after immunoprecipitation with control IgG (Fig. 5e).

The data above showed that STAT3 mediates PDK1 transcription through a region of 279 nucleotides from − 410 to − 131 that has four putative SRE (Fig. 5b). Following a further analysis of this region using oPOSSUM [33], we narrowed down the candidate sites to the ones starting at − 265 and − 137, which displayed the highest scores. To precisely identify the functional SREs on the PDK1 promoter, the reporter assays were performed using the − 410/+ 82 vectors harboring mutations that destroyed the SREs at − 265 and − 137 sites of the PDK1 promoter. Mutation of either of these sites reduced the reporter activity in UACC903-scrambled cells compared to the wild-type promoter (Fig. 5f). In line with this data, the simultaneous mutation of both sites drastically reduced the luciferase activity (Fig. 5f). Silencing of STAT3 further reduced the activity of each promoter fragment indicating that the mutant reporters are still transactivated by STAT3. In contrast, the activity driven by the double mutant promoter was similar in both UACC903-scramble and UACC903-shSTAT3 cells indicating that this promoter fragment had completely lost STAT3 responsiveness (Fig. 5f). Altogether, these results indicate that STAT3 transactivates PDK1 through two SRE located at − 265 and − 137. Interestingly, neither of the two SRE displays the canonical sequence TTCN^2–4^GAA. However, the site at − 265 (TGCCGGAA) displays two half-sites (TGC and GAA) that are identical to the ones described on the SRE of IL10 [34], GADD45B [35] and STAT3 [36]. The site at -137 (AACCCGGAA) present similarities with the SRE of LBP (CACTGGGAA, one mismatch on the 5′ half-site, [37]) and with two SRE of Gamma Fibrinogen (site II, ATCGGCGAA and site III, GACTGGGAA, both displaying one mismatch on the 5′ half-site, [38]). Taken together, these results demonstrate a direct interaction between activated STAT3 and regulatory elements of the PDK1 gene and suggest direct transcriptional regulation of PDK1 by STAT3.

### PDK1 partly rescues cell death triggered by dacarbazine treatment and STAT3 silencing

**Fig. 6:**
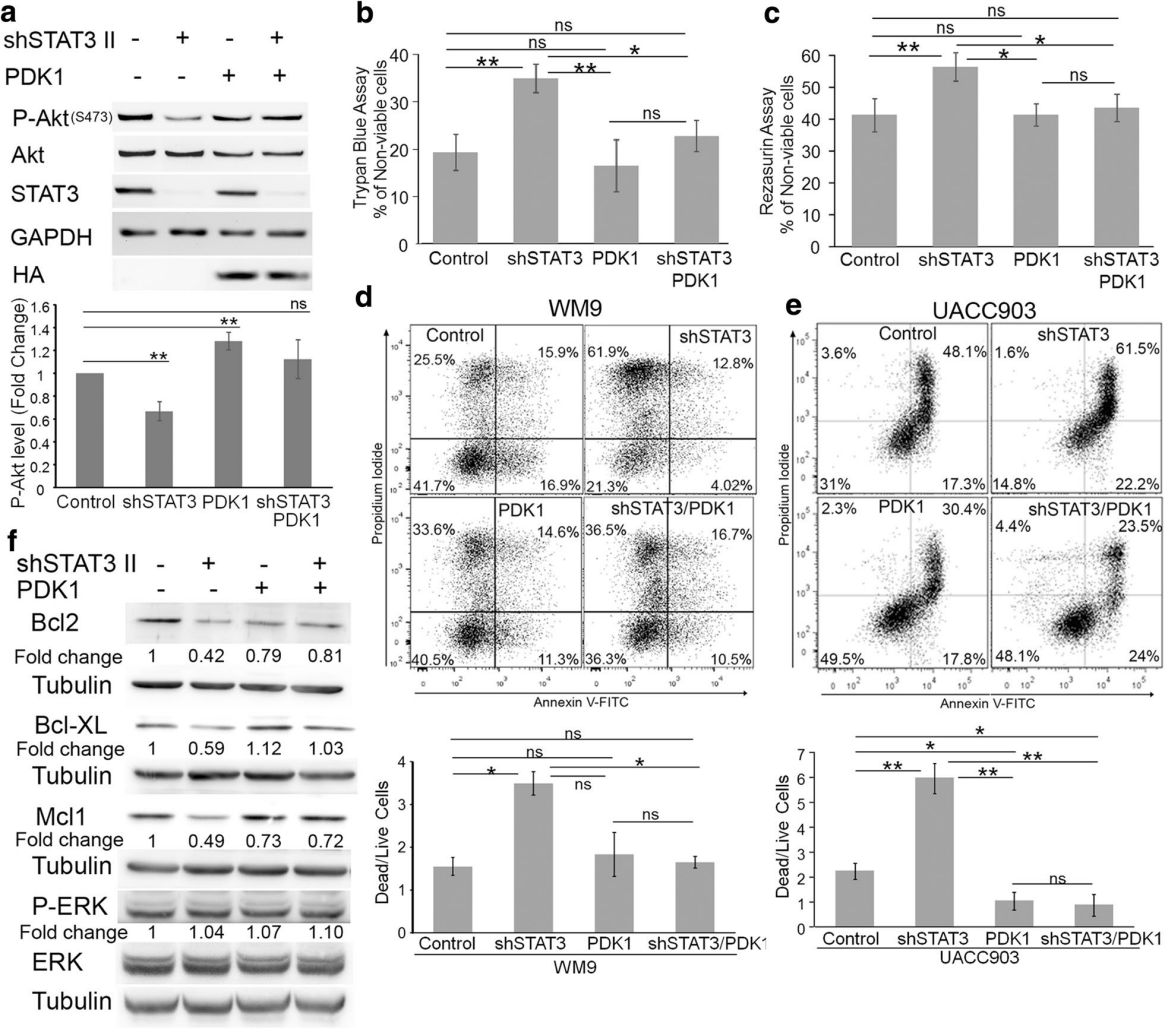
PDK1 mediates STAT3’s effect on melanoma cell survival. **a** Reconstitution of PDK1 upon STAT3 silencing rescued Akt phosphorylation. WM9-scramble and WM9-shSTAT3 cells were stably transduced with HA-PDK1. Protein extracts were probed with STAT3, P-Akt, Akt, HA and GAPDH as a loading control. The blots displayed are representative of three independent experiments. Bar graphs show the mean ± S.D. (from three independent experiments) of P-Akt levels normalized to total Akt and expressed as the fold change relative to scramble/pBH cells (Control). Statistical significance was tested by one-tailed Student’s T-Test using log transformed FC values. **p < 0.001, ns: not significant, n = 5. **b**–**e** PDK1 reconstitution prevents dacarbazine-induced cell death in STAT3 knockdown cells. The cells were treated with dacarbazine (100 μg/ml) for 72 h. **b** Viability was determined by the Trypan Blue exclusion assay. Bar Graph shows the mean ± S.D. (from three independent experiments) of the percentage of non-viable cells. Statistical significance was tested by ANOVA followed by Tukey’s Multiple Comparison Test. *p < 0.01 **p < 0.001, ns: not significant, n = 3. **c** Viability was determined by the Resazurin assay. Bar Graph shows the mean ± S.D. (from three independent experiments) of the percentage of non-viable cells. Statistical significance was tested by ANOVA followed by Tukey’s Multiple Comparison Test. *p < 0.01 **p < 0.001, ns: not significant, n = 3. **d**, **e** WM9 (**d**) or UACC903 (**e**) cells were treated with dacarbazine, stained with PI/Annexin V and analyzed by Flow cytometry. Representative histograms are shown. The ratio of dead/live cells from three biological replicates ± S.D is shown below. Live cells correspond to cells in the lower left quadrant and dead cells are the sum of the other three quadrants. Statistical significance was tested by ANOVA followed by Tukey’s Multiple Comparison Test. *p < 0.01 **p < 0.001, ns: not significant, n = 3. **f** PDK1 mediates STAT3’s effect on expression of anti-apoptotic proteins. Protein extracts from WM9-scramble and WM9-shSTAT3 cells stably transduced with PDK1 were blotted with the indicated antibodies. Tubulin was used as loading control. Protein levels were normalized to the corresponding loading control and expressed as the fold change (numbers below the blots) relative to scramble cells

We next proceeded to address the biological significance of the STAT3-PDK1 connection. To this end, we rescued the expression of PDK1 in STAT3 knockdown cells by stable transducing WM9-scramble and WM9-shSTAT3 cells with a retrovirus expressing HA-tagged PDK1 or empty vector (Fig. 6a). As shown before, STAT3 silencing significantly inhibited S473 Akt phosphorylation (lane 2). Expression of HA-PDK1 increased P-Akt levels in scramble cells (lane 3) and restored P-Akt levels in STAT3 knockdown cells (lane 4) to levels slightly above to those of control cells (lane 1)(Fig. 6a).

In recent years, several articles have pointed out the important role of STAT3 inhibition in apoptosis in melanoma [39–41]. However, STAT3 silencing did not altered neither cell proliferation (Additional file 2: Figure S2) nor cell death under normal growing conditions (see below) likely due to pathway redundancy. For the STAT3’s role in apoptosis to materialize, additional events are required. One of such events is a chemotherapy treatment [42, 43]. Since dacarbazine has been shown to promote a relatively weak apoptotic response in melanoma [44] we speculated that combining this cytotoxic insult with STAT3 silencing could be a good model to assess the contribution of the STAT3/PDK1 pathway on cell death. In the first place, we evaluated the effect of dacarbazine by assessing cell viability using the Trypan Blue exclusion method. As shown in Fig. 6b, dacarbazine treatment induced a significant increase in the number of non-viable cells in WM9-shSTAT3 cells compared with WM9-scramble cells. This increase was prevented by expression of HA-PDK1 (Fig. 6b). These findings were confirmed by using the Resazurin reduction assay (Fig. 6c). Next, we assessed apoptotic cell death through PI/Annexin V labeling and subsequent flow cytometry analysis. Silencing of STAT3 was not sufficient to induce cell death in WM9 cells under normal growing conditions (Additional file 3: Figure S3) but significantly increased the amount of dead cells (which corresponds to the sum of apoptotic plus necrotic cells) compared to WM9-scramble cells upon dacarbazine treatment (Fig. 6d). Importantly, this increased mortality was prevented by HA-PDK1 expression (Fig. 6d). To confirm these findings we expressed PDK1 in both UACC903 scramble and STAT3 knockdown cells as was described for WM9. Again, PDK1 expression significantly blocked the cell death induced by STAT3 silencing upon dacarbazine treatment (Fig. 6e). Unlike WM9, UACC903 cells expressing PDK1 presented a more resistant phenotype than control cells (Fig. 6e). Taken together, these results demonstrate that cell death triggered by dacarbazine and facilitated by STAT3 depletion is mediated, at least in part, by PDK1 and downstream pathways.

One of the critical mechanisms by which STAT3 mediates apoptosis resistance is the upregulation of several anti-apoptotic members of the Bcl-2 family [16]. To evaluate the involvement of PDK1 in the STAT3-dependent regulation of anti-apoptotic proteins we evaluated the expression of Bcl2, Mcl1 and Bcl-XL in WM9 cells after STAT3 silencing and PDK1 re-expression. The expression levels of Bcl2, Mcl1 and Bcl-XL were reduced upon STAT3 silencing compared to control cells (Fig. 6f, compare lane 1 and 2). As shown in Fig. 6f, expression of HA-PDK1 restored the expression of the three STAT3 targets down-regulated by STAT3 silencing (compare lane 2 and 4). The effect was pronounced on Bcl-XL and partial on Bcl-2 and Mcl1 reflecting slight differences on their regulation by both STAT3 and PDK1 signals. Importantly, manipulation of either STAT3 or PDK1 did not affect the activity of ERK/MAPK, a signaling pathway that has also being implicated in control of Bcl2 family protein expression [45]. Taken together, these findings provide direct evidence that PDK1 is required for STAT3’s effect on apoptosis by regulating the expression of the anti-apoptotic proteins Bcl2, Bcl-XL and Mcl1.

## Discussion

The potential benefits of inhibiting STAT3 signaling have long been recognized in the cancer field [46]. Unfortunately, in contrast to the great success of tyrosine kinase inhibitors, development of drugs targeting transcription factors including STAT3 has lagged behind, mainly due to the greater complexity implicated in disrupting protein–protein or protein–DNA interactions. Recent pieces of evidence have shown a new facet of STAT3 in tumorigenesis emphasizing its status as a promising therapeutic target. Work by Liu et al. [11] and Girotti et al. [12] in melanoma have shown that STAT3 activation is a new mechanism of resistance to vemurafenib treatment. In addition, Sos et al. showed that an autocrine IL-6/JAK/STAT3 pathway contributes to the intrinsic vemurafenib-resistant phenotype seen in many BRAF-mutant cells [14]. Vultur et al. also showed STAT3 activation in resistant cells and revealed that targeting the STAT3 pathway prevents the invasive phenotype induced by MEK inhibition [13]. Interestingly, STAT3 activation seems to be a general mechanism of acquired resistance since it was also observed in cancer cells driven by diverse kinases, including EGFR, HER2, ALK, and MET, as well as mutant KRAS following treatment with specific inhibitors [47]. In this context, it is anticipated that an in-depth understanding of STAT3 pathway, its downstream targets and their contribution to cancer progression could allow for improved therapeutic agents and interventions.

In the current study, we established that STAT3 is constitutively bound to the PDK1 promoter and promotes PDK1 transcription in melanoma through at least two STAT3 responsive elements. This regulation most likely play a particularly relevant role in the altered context of a tumor cell by strengthening the phosphorylation and activation of AGC kinases including Akt, PKC, and SGK. Indeed, overexpression of PDK1 due to an increase in gene copy number or protein overexpression have been demonstrated in breast cancer and acute myeloid leukemia, among other malignancies [48] and can be of critical importance to boost the signal output of upstream alterations such as PTEN, PIK3CA and ERBB2 to Akt [49].

The data presented here provide a mechanistic explanation for the well-documented functional association observed between the STAT3 and PI3K/Akt signaling pathways in cancer [50]. STAT3 was shown to positively regulate Akt phosphorylation [51, 52] and indirect evidence has also linked STAT3 to PDK1. Xiao et al. have described that atractylenolide-1, a bioactive herb compound from traditional Chinese herbs with anti-inflammatory and anti-tumor activities, stimulates ERK phosphorylation and inhibited phosphorylation and protein expression of STAT3, SP1 and PDK1 in lung cancer cells [53]. Similarly, the candidate antimetastatic agent 6BIO, an indirubin derivative, was shown to inhibit phosphorylation of STAT3, GSK3 and Thr^308^ phosphorylation of Akt (the site phosphorylated by PDK1) [54]. Similarly, an in vivo model of squamous cell carcinoma driven by constitutive activation of Src showed increased levels of activated PDK1, STAT3, and ERK1/2 in the lesioned tissue [55]. However, none of these studies have elucidated the precise link between STAT3 and PDK1.

Our results showed that STAT3 silencing by itself does not significantly affect neither proliferation nor cell death of WM9 melanoma cells likely because these cells harbor both BRAFV600E mutation and PTEN homozygous deletion. However, in the presence of dacarbazine, STAT3 silencing significantly increased cell death. Notably, overexpression of PDK1 markedly reduced dacarbazine sensitivity induced by STAT3 shRNA. Our data show that PDK1 overexpression rescued the expression of Bcl-2, Bcl-xL y Mcl-1 whose levels were down-regulated upon STAT3 silencing. This result is in agreement with data from Pugazhenthi et al. that showed a PDK1-dependent increase in Bcl2 promoter activity in PC12 cells [56]. These results indicate that STAT3 can regulate the expression of Bcl2 family proteins both directly (by transactivating them) and indirectly by modulating PDK1 transcription. PDK1, in turn, regulates the activity of AGC kinases that could phosphorylate and engage other transcription factors in the expression of Bcl2 family proteins. It has been documented that Akt regulates Bcl2 and Bcl-XL expression by activating CREB [57] and NF-κB respectively [57] and SGK1 regulates Mcl-1 expression through STAT1/STAT2 [58].

This study has established that STAT3 regulates PDK1 and have a profound impact on PDK1 downstream signaling pathways. The constitutive activity of STAT3 in melanoma contributes to increase the expression level of PDK1 which, in turn phosphorylate and activate AGC kinases including Akt, PKC, and SGK. These finding provide a mechanistic explanation for the association observed in cancer between STAT3 and PI3K/Akt pathway. Our results also established that PDK1 is an important mediator of drug-resistance mediated by STAT3 in melanoma. These results add further support to both STAT3 and PDK1 as rational targets to complement either BRAF-targeted therapy in melanoma or chemotherapy in other tumor types.

## Additional files

**Additional file 1.** STAT3β inhibits the luciferase activity driven by PDK1 promoter.

**Additional file 2.** Neither STAT3 silencing nor PDK1 overexpression affects WM9 cell proliferation.

**Additional file 3.** Neither STAT3 silencing nor PDK1 overexpression induces changes in apoptosis.

## Abbreviations

PDK1: phosphoinositide-dependent kinase 1
STAT3: Signal Transducer and Activator of Transcription 3
Akt: RAC-alpha serine/threonine-protein kinase
SRE: STAT3 responsive elements
GAPDH: glyceraldehyde-3-phosphate dehydrogenase
PI: propidium iodide
PI3K: phosphoinositide-3-kinase
RTK: receptor tyrosine kinases
qRT-PCR: quantitative reverse transcriptase-polymerase chain reaction
shRNA: short hairpin RNA
RNPII: RNA polymerase II
