# Supplementary figures and images for: STAT3 enhances the constitutive activity of AGC kinases in melanoma by transactivating PDK1
Source: Cell Biosci. 2019 Jan 3;9:3. doi: 10.1186/s13578-018-0265-8 (PMC6317239; doi:10.1186/s13578-018-0265-8)

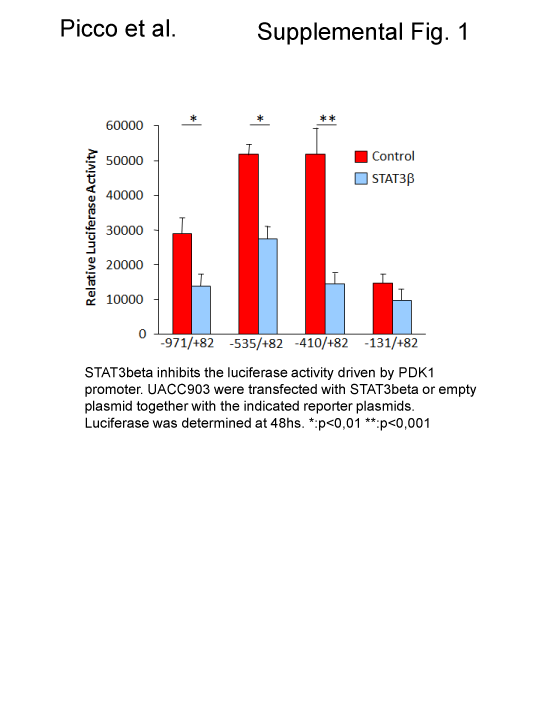

Supplement: Supplementary file 1 — Additional file 1. STAT3β inhibits the luciferase activity driven by PDK1 promoter. [file 13578_2018_265_MOESM1_ESM.tif]

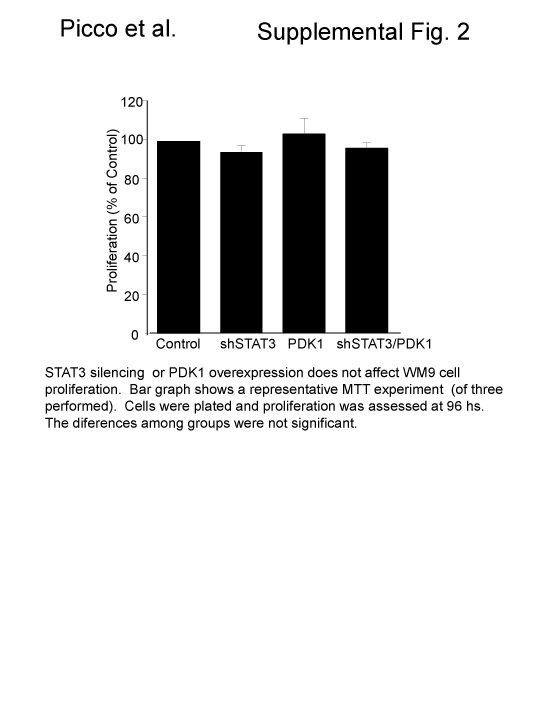

Supplement: Supplementary file 2 — Additional file 2. Neither STAT3 silencing nor PDK1 overexpression affects WM9 cell proliferation. [file 13578_2018_265_MOESM2_ESM.tif]

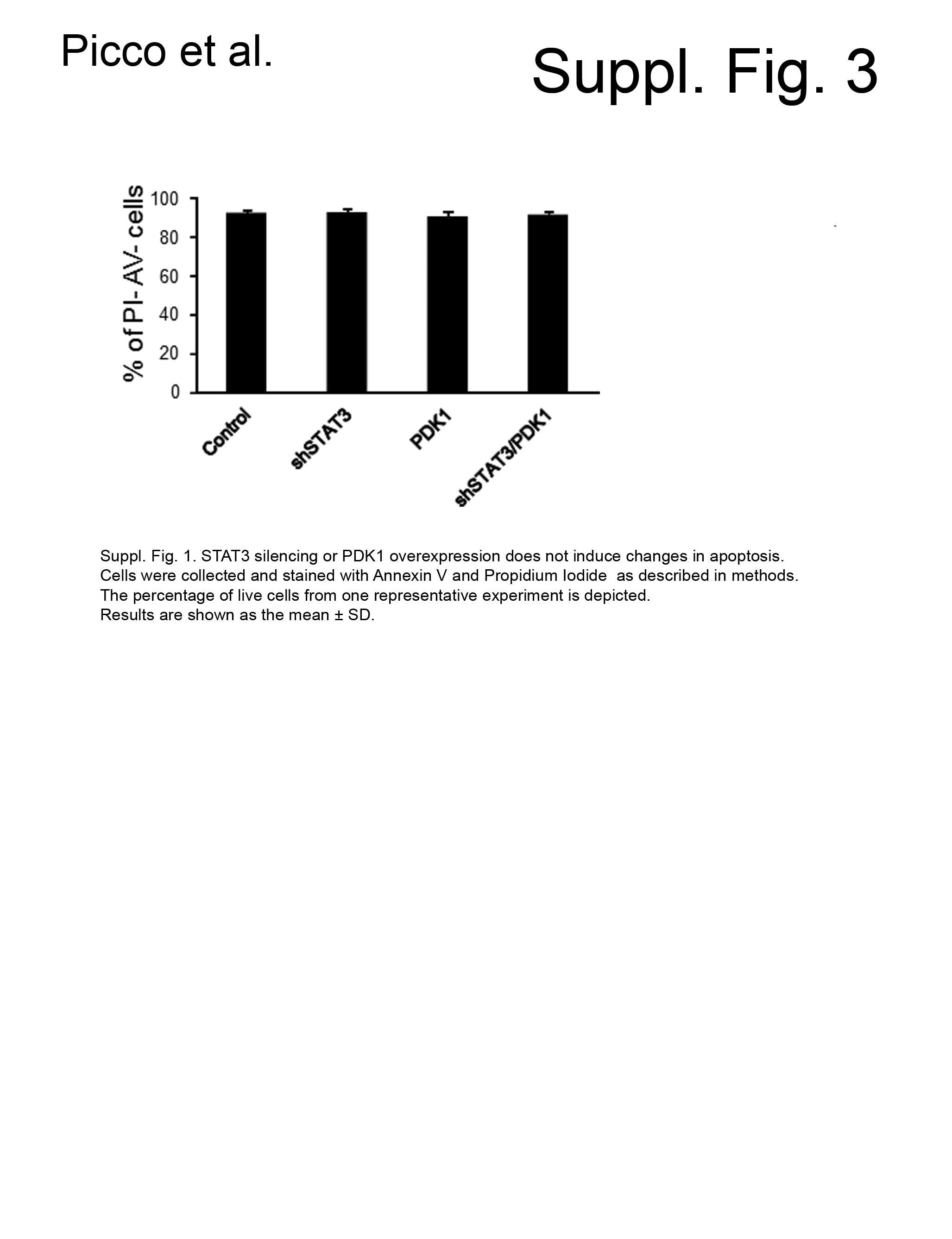

Supplement: Supplementary file 3 — Additional file 3. Neither STAT3 silencing nor PDK1 overexpression induces changes in apoptosis. [file 13578_2018_265_MOESM3_ESM.tif]
